# Supplementary figures and images for: Condensate-forming eIF4ET ensures adequate levels of meiotic proteins to support oocyte storage
Source: Life Sci Alliance. 2025 May 29;8(8):e202503387. doi: 10.26508/lsa.202503387 (PMC12122253; doi:10.26508/lsa.202503387)

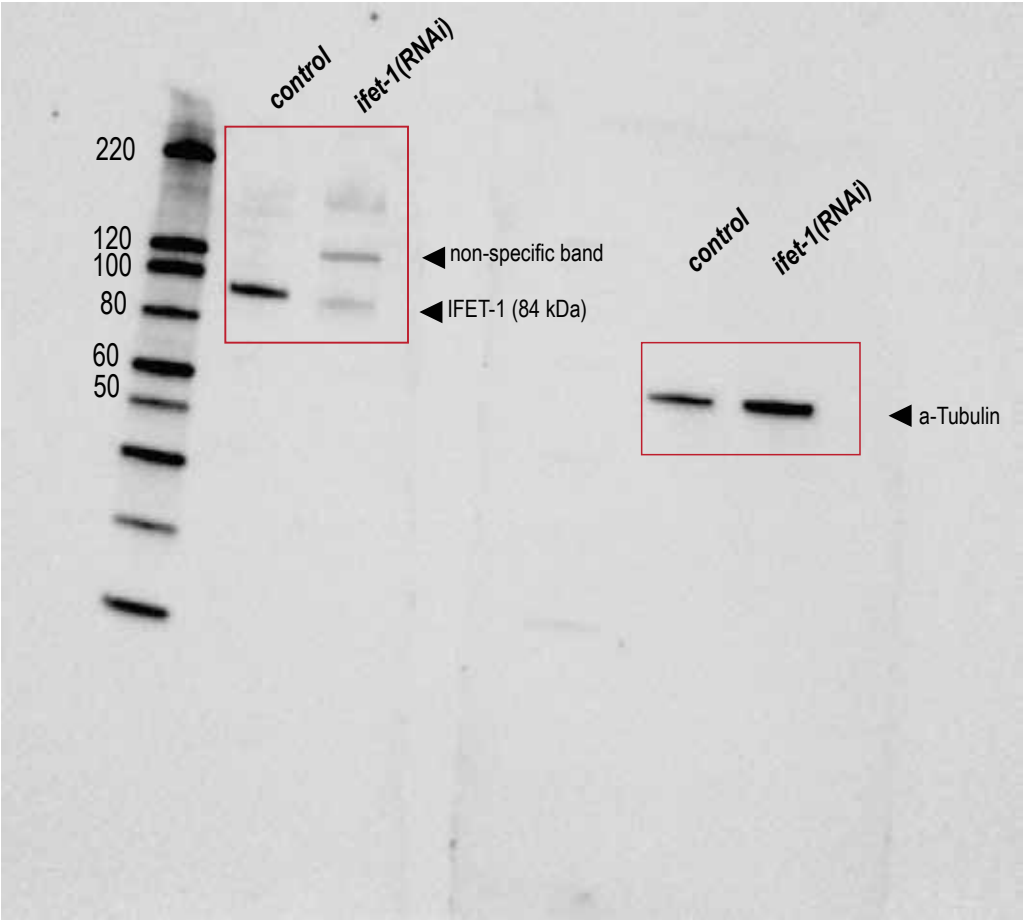

Supplement: Supplementary file 2 [file LSA-2025-03387_SdataFS1.pdf]

WB: IFET-1

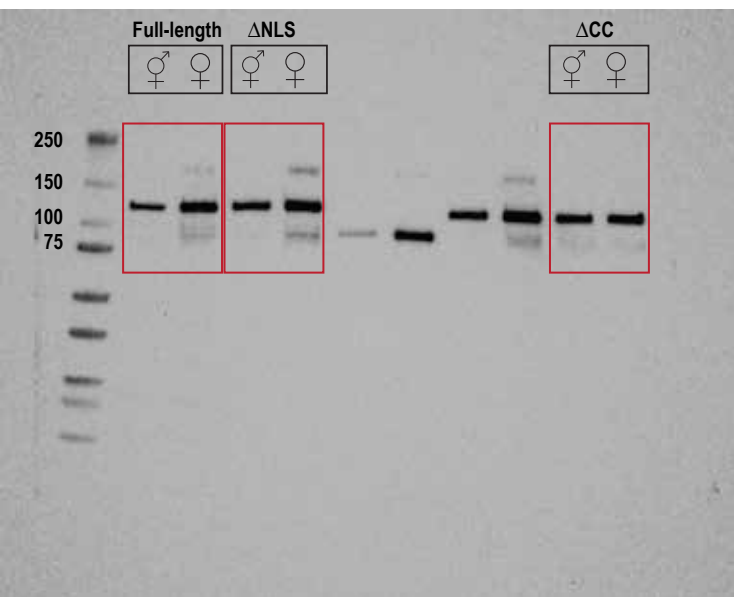

Ponceau

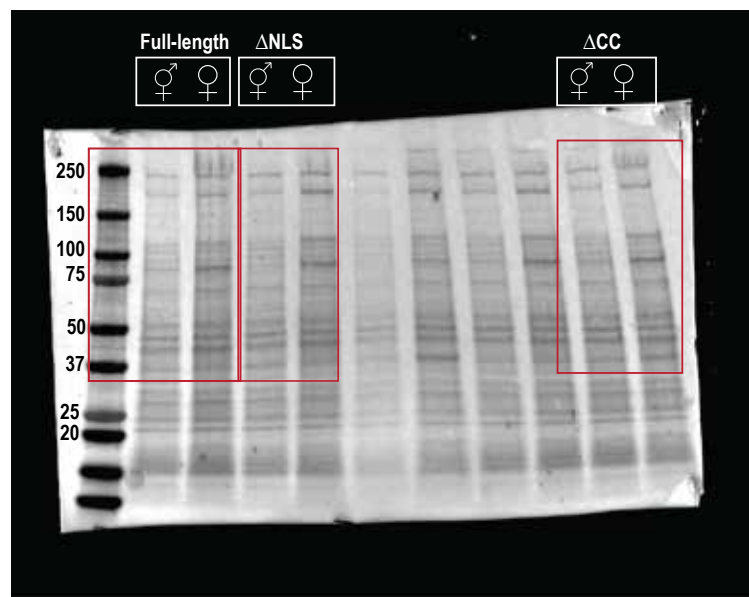

WB: IFET-1

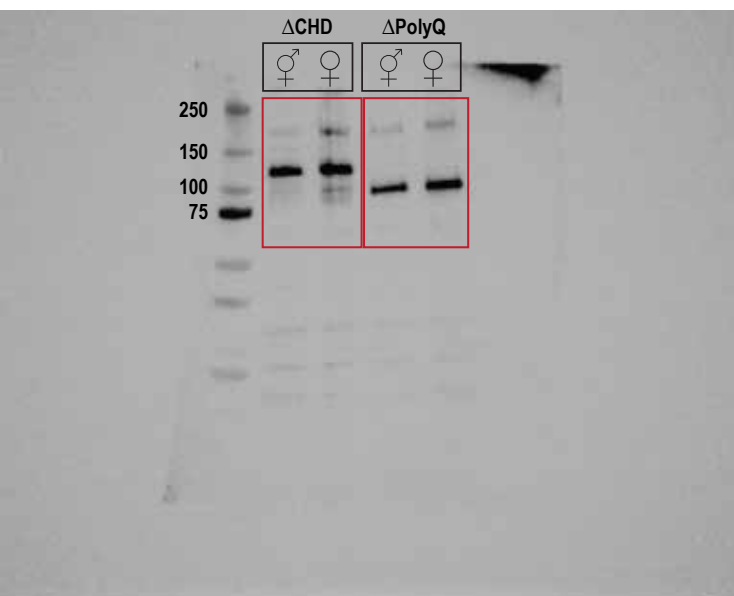

Ponceau

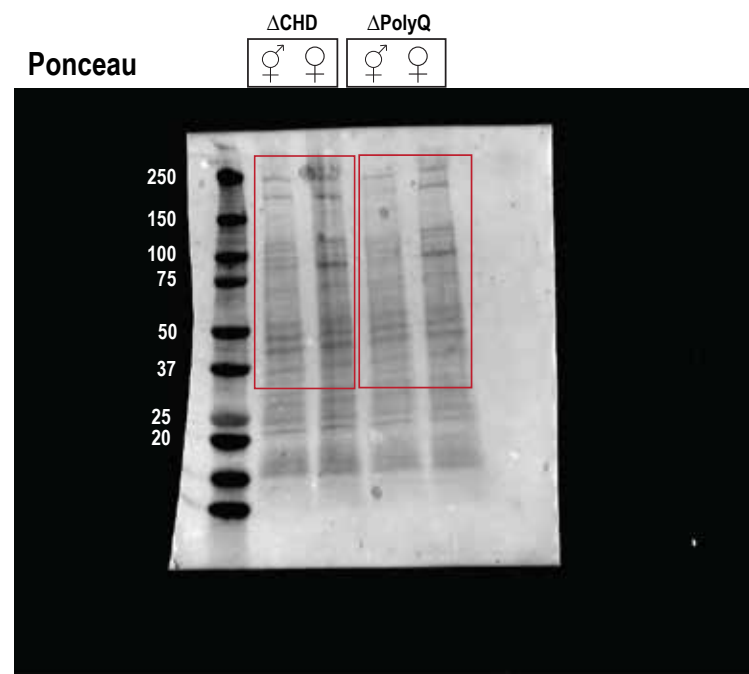

Supplement: Supplementary file 3 [file LSA-2025-03387_SdataFS2.pdf]

## Puromycin

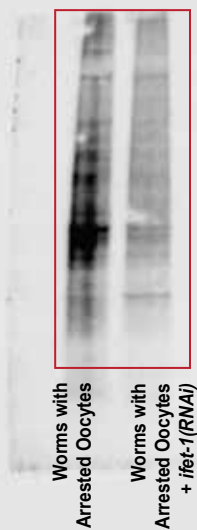

## Ponceau

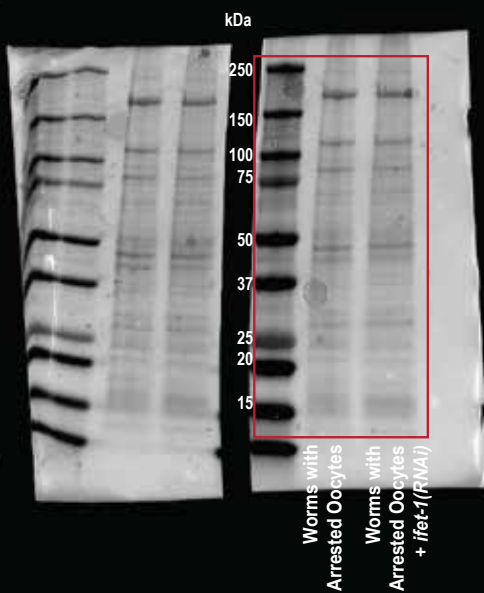

Supplement: Supplementary file 6 [file LSA-2025-03387_SdataF3.pdf]

# Puromycin

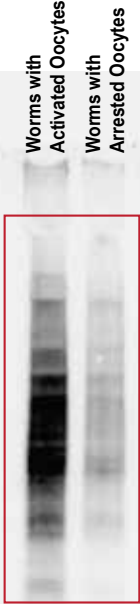

# Ponceau

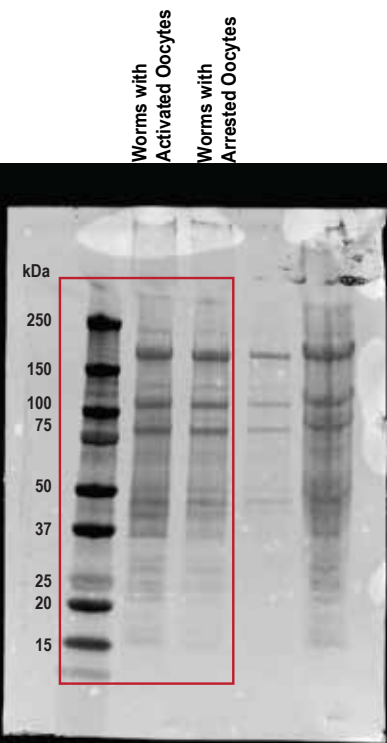

Supplement: Supplementary file 8 [file LSA-2025-03387_SdataFS3.pdf]
