# Supplementary material for: Condensate-forming eIF4ET ensures adequate levels of meiotic proteins to support oocyte storage
Source: Life Sci Alliance. 2025 May 29;8(8):e202503387. doi: 10.26508/lsa.202503387 (PMC12122253; doi:10.26508/lsa.202503387)
Supplement: Supplementary file 9 [file LSA-2025-03387_TableS1.docx]

Table S1. *C. elegans* strains.

| <!--Col Count:4-->Strain name | genotype | Creation method | Origin |
| --- | --- | --- | --- |
| BA17 | *fem-1 (hc17) IV* | mutagenesis | CGC |
| CB4108 | *fog-2(q71) V* | mutagenesis | CGC |
| JWW112 | *car-1(utsw6[car-1::mMaple]) I* | CRISPR | This study |
| JWW166 | *ifet-1(utsw15[ifet-1::mMaple]) III* | CRISPR | This study |
| JWW173 | *utsw15[ifet-1::mMaple]III; fem-1 (hc17) IV* | cross | This study |
| JWW140 | *car-1(utsw6[car-1::mMaple]) I; fem-1(hc17) IV* | cross | This study |
| JWW236 | *mei-1(utsw17[gfp::mei-1]) I; fem-1 (hc17) IV* | CRISPR | This study |
| JWW163 | *[pie-1p::GFP::H2B + unc-119(+)] III;fem-1 (hc17) IV* | cross | This study |
| PHX6862 | *ifet-1(syb6862[ifet-1(∆196-217)::mMaple]) III* | CRISPR | SunyBiotech |
| JWW228 (∆CHD) | *ifet-1(syb6862[ifet-1(∆196-217)::mMaple]) III; fem-1(hc17) IV* | cross | This study |
| PHX6949 | *ifet-1(syb6949[ifet-1(∆220-235)::mMaple]) III* | CRISPR | SunyBiotech |
| JWW215 (∆NLS) | *ifet-1(syb6949[ifet-1(∆220-235)::mMaple]) III; fem-1(hc17) IV* | cross | This study |
| PHX6886 | *ifet-1(syb6862[ifet-1(∆527-644)::mMaple]) III* | CRISPR | SunyBiotech |
| JWW216 (∆polyQ) | *ifet-1(syb6862[ifet-1(∆527-644)::mMaple]) III; fem-1(hc17) IV* | cross | This study |
| PHX6954 | *ifet-1(syb6862[ifet-1(∆664-691)::mMaple]) III* | CRISPR | SunyBiotech |
| JWW230 (∆CC) | *ifet-1(syb6862[ifet-1(∆664-691)::mMaple]) III; fem-1(hc17) IV* | cross | This study |
| JWW252 | *fem-1 (hc17) IV; ruls57[pie-1p::GFP::tubulin + unc-119(+)]* | cross | This study |
| JWW253 | *ifet-1(utsw16[ifet-1::mScarlet_I::AID*::3xFlag]) III; wrdSi3 [sun-1p::TIR1::F2A::mTagBFP2::AID*::NLS::tbb-2 3'UTR] (II:0.77).* | CRISPR | This study |
| JWW255 | *fem-1 (hc17) IV; ifet-1(utsw16[ifet-1::mScarlet_I::AID*::3xFlag]) III; wrdSi3 [sun-1p::TIR1::F2A::mTagBFP2::AID*::NLS::tbb-2 3'UTR] (II:0.77).* | cross | This study |
